# Violence against healthcare workers during COVID-19 vaccination campaign

**DOI:** 10.1093/eurpub/ckac131.266

**Published:** 2022-10-25

**Authors:** T Lo Presti, E Scarpis, L Arnoldo, F Fiorillo, P Zuliani, F Farneti, E Croci, B Pellizzari, R Cocconi, L Brunelli

**Affiliations:** 1 Department of Medicine, University of Udine, Udine, Italy; 2 Quality and Clinical Risk Management Unit, Friuli Centrale Healthcare University Trust, Udine, Italy; 3 Regional Transplant Center, Friuli Centrale Healthcare University Trust, Udine, Italy; 4 Department of Prevention, Giuliano Isontina Healthcare University Trust, Trieste, Italy; 5 Department of Prevention, Friuli Occidentale Healthcare Trust, Pordenone, Italy

## Abstract

**Background:**

Violence against healthcare workers (HCWs) has gained increasing attention in recent years, both because it is on the rise and because there is growing concern about these incidents, which are classified as sentinel events by the Italian Ministry of Health. Since little is known about the actual burden of this phenomenon, especially during the stressful COVID-19 vaccination campaign, we decided to explore the problem in our region.

**Methods:**

Between January and March 2022, in the 1.2 million-inhabitant Friuli Venezia Giulia region (Italy), we conducted an anonymous online survey to collected data on episodes of violence and their consequences for HCWs. Data from validated tools assessing characteristics of violent episodes and post-trauma impact were analyzed in conjunction with socio-demographic data of respondents.

**Results:**

200 HCWs participated in the survey, most of whom were women (72%), worked as nurses (107) or doctors (71), and had a mean age of 47 years. More than half of them (59%) reported at least one episode of violence, mainly in the form of a verbal assault (64%); there were no differences between victims. In 72 cases, these incidents affected the HCW’s private life, mainly disturbing sleep or concentration (68%). Most HCWs stated that inadequate communication was a trigger for the violence (97%). Although 80% of HCWs would readily report any violent incident, to improve the situation they called for certainty of action against the perpetrator (87%), more organizational support (85%), standard procedures (75%) and self-defense courses (75%).

**Conclusions:**

Violence in COVID-19 vaccination campaign appears to be common and to affect both the personal and professional lives of HCWs. Improvements at the institutional and personal level could help to address this problem that affects the health workforce.

**Key messages:**

• Given the growing public debate, violence in healthcare settings is an important issue that needs to be addressed in the coming years.

• Assessing workplace violence and its associated risk factors will help focus on the strategies that can be usefully employed to prevent it in the future.

